# A Membrane‐Targeting Photosensitizer with Aggregation‐Induced Emission Characteristics for Highly Efficient Photodynamic Combat of Human Coronaviruses

**DOI:** 10.1002/smll.202101770

**Published:** 2021-06-30

**Authors:** Ming‐Yu Wu, Meijia Gu, Jong‐Kai Leung, Xinmei Li, Yuncong Yuan, Chao Shen, Lianrong Wang, Engui Zhao, Sijie Chen

**Affiliations:** ^1^ Key Laboratory of Combinatorial Biosynthesis and Drug Discovery Ministry of Education School of Pharmaceutical Sciences Wuhan University Wuhan Hubei 430071 China; ^2^ Ming Wai Lau Centre for Reparative Medicine Karolinska Institutet Hong Kong 999077 China; ^3^ School of Science Harbin Institute of Technology Shenzhen HIT Campus of University Town Shenzhen 518055 China; ^4^ College of Life Sciences and China Center for Type Culture Collection Wuhan University Wuhan Hubei 430071 China; ^5^ School of Life Science and Engineering Southwest Jiaotong University Chengdu 610031 China

**Keywords:** aggregation‐induced emission, human coronaviruses, membrane targeting, photodynamic therapy, photosensitizers

## Abstract

COVID‐19 pandemic, caused by severe acute respiratory syndrome coronavirus 2, has resulted in global social and economic disruption, putting the world economy to the largest global recession since the Great Depression. To control the spread of COVID‐19, cutting off the transmission route is a critical step. In this work, the efficient inactivation of human coronavirus with photodynamic therapy (PDT) by employing photosensitizers with aggregation‐induced emission characteristics (DTTPB) is reported. DTTPB is designed to bear a hydrophilic head and two hydrophobic tails, mimicking the structure of phospholipids on biological membranes. DTTPB demonstrates a broad absorption band covering the whole visible light range and high molar absorptivity, as well as excellent reactive oxygen species sensitizing ability, making it an excellent candidate for PDT. Besides, DTTPB can target membrane structure, and bind to the envelope of human coronaviruses. Upon light irradiation, DTTPB demonstrates highly effective antiviral behavior: human coronavirus treated with DTTPB and white‐light irradiation can be efficiently inactivated with complete loss of infectivity, as revealed by the significant decrease of virus RNA and proteins in host cells. Thus, DTTPB sensitized PDT can efficiently prevent the infection and the spread of human coronavirus, which provides a new avenue for photodynamic combating of COVID‐19.

## Graphical abstract

In this work, we report the application of a novel photosensitizer, DTTPB, with aggregation‐induced emission characteristics for photodynamic inactivation of human coronaviruses. With high molar absorbance coefficient, broad absorption band covering the whole white light region, and high membrane targeting and ROS sensitizing efficiency, DTTPB could effectively inactivate human coronaviruses and may contribute to preventing the spread of COVID‐19 pandemic.

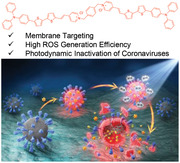

## 1 Introduction

In 2020, we have witnessed the waging of COVID‐19, caused by the widespread of severe acute respiratory syndrome coronavirus 2 (SARS‐CoV‐2).^[^ ^1^, ^2^, ^3^ ^]^ So far, COVID‐19 has caused more than three million death worldwide (data was from Johns Hopkins University till 26/04/2021), and resulted in a profound influence on social and economic development. The COVID‐19 pandemic reminds us that human beings are still not well prepared in face of virus outbreaks, despite the simple structure of the virus as compared with mammalian cells. This could be partially ascribed to the high frequency of virus mutation.^[^ ^4^ ^]^ The mutation rate in RNA virus, such as human coronavirus, is even higher than DNA virus, which makes the development of vaccines and treatments very challenging.^[^ ^5^, ^6^ ^]^

Cutting off the virus spreading route is an effective approach for thwarting virus outbreaks.^[^ ^7^ ^]^ Taking COVID‐19 pandemic with more than 147 million reported cases (data was from Johns Hopkins University till 26/04/2021) as an example, its widespread, to a considerable extent, was facilitated by SARS‐CoV‐2 contaminated high‐touch surfaces.^[^ ^8^ ^]^ Great efforts were spent to prevent virus transmission by killing and/or reducing the attachment of microbes. Though disinfectants, such as ethanol and hypochlorite, were frequently sprayed onto these high‐touch surfaces for inactivating virus, their frequency should be maintained at a high level, due to the rapid evaporation or deactivation of these disinfectants.^[^ ^9^ ^]^ Therefore, developing efficient surfaces and coatings materials to minimize the presence of active viral pathogens and prevent the spread of infectious pathogens in a variety of public places, such as hospitals, public transportations, and schools, are highly desirable.^[^ ^10^, ^11^, ^12^ ^]^

As a prominent therapeutic strategy for contemporary and cost‐effective precision medicine, photodynamic therapy (PDT) has attracted more and more attention in antibiosis and tumor therapy.^[^ ^13^, ^14^, ^15^, ^16^, ^17^ ^]^ In PDT, photosensitizers (PSs) and light irradiation were employed to sensitize the generation of reactive oxygen species (ROS), which can cause oxidative damages to nucleic acids, proteins, or lipids.^[^ ^18^ ^]^ Consequently, it induces irreversible microbe deaths with the superiority of minimal invasiveness, limited antibiotic resistance, low systemic toxicity, and minimal side effects.^[^ ^19^ ^]^ Photodynamic inactivation (PDI) of microbes has received considerable attention from scientists since the beginning of the 20th century.^[^ ^20^ ^]^ Recently, a resurgent on employing PDT for combating bacteria and fungi was observed.^[^ ^21^ ^]^ Besides, photodynamic antiviral studies also start to gain increasing attention, since Dr. Nicholas Kipshidze proposed “photodynamic therapy for COVID‐19” in Nature Photonics in 2020.^[^ ^22^, ^23^ ^]^

The outcome of PDT is closely related to the PS employed: effective PSs with high target specificity and ROS sensitizing efficiency are highly favorable for PDT applications.^[^ ^24^ ^]^ Conventional PSs, such as phenothiazinium salts, porphyrins, phthalocyanines, diketopyrrolopyrroles, and cyanines, have been widely employed for photodynamic anticancer and antibacterial studies. However, these PSs are usually featured with coplanar structures with large π conjugations, and tend to experience strong π‐π interactions in the aggregated state or at high concentrations, which could decay excited‐state energy through non‐radiative pathways, and result in a drastic decrease in both fluorescence (FL) intensity and ROS sensitizing efficiency.^[^ ^25^ ^]^ The low ROS sensitizing efficiency in the aggregated states is detrimental for applications in high‐touch surface disinfections, as well as fabrication into particles for PDT.^[^ ^26^ ^]^ Though the issue could be solved by introducing bulky groups to prevent π‐π interactions, it usually significantly increases the synthetic difficulties.^[^ ^27^, ^28^ ^]^ PSs with aggregation‐induced emission (AIE) properties have recently become powerful tools for PDT of bacteria, fungi, and tumor.^[^ ^21^, ^24^, ^26^, ^29^ ^]^ These PSs are usually featured with propeller‐shaped molecular conformations, and show not only enhanced FL but also elevated ROS sensitizing efficiency in the aggregated or solid‐state, as compared to that in the discrete molecular state. Despite the successful applications of AIEgen for PDT of bacteria, fungi, and tumor, research on PDI of the virus has been seldom reported.

**Figure 1:**
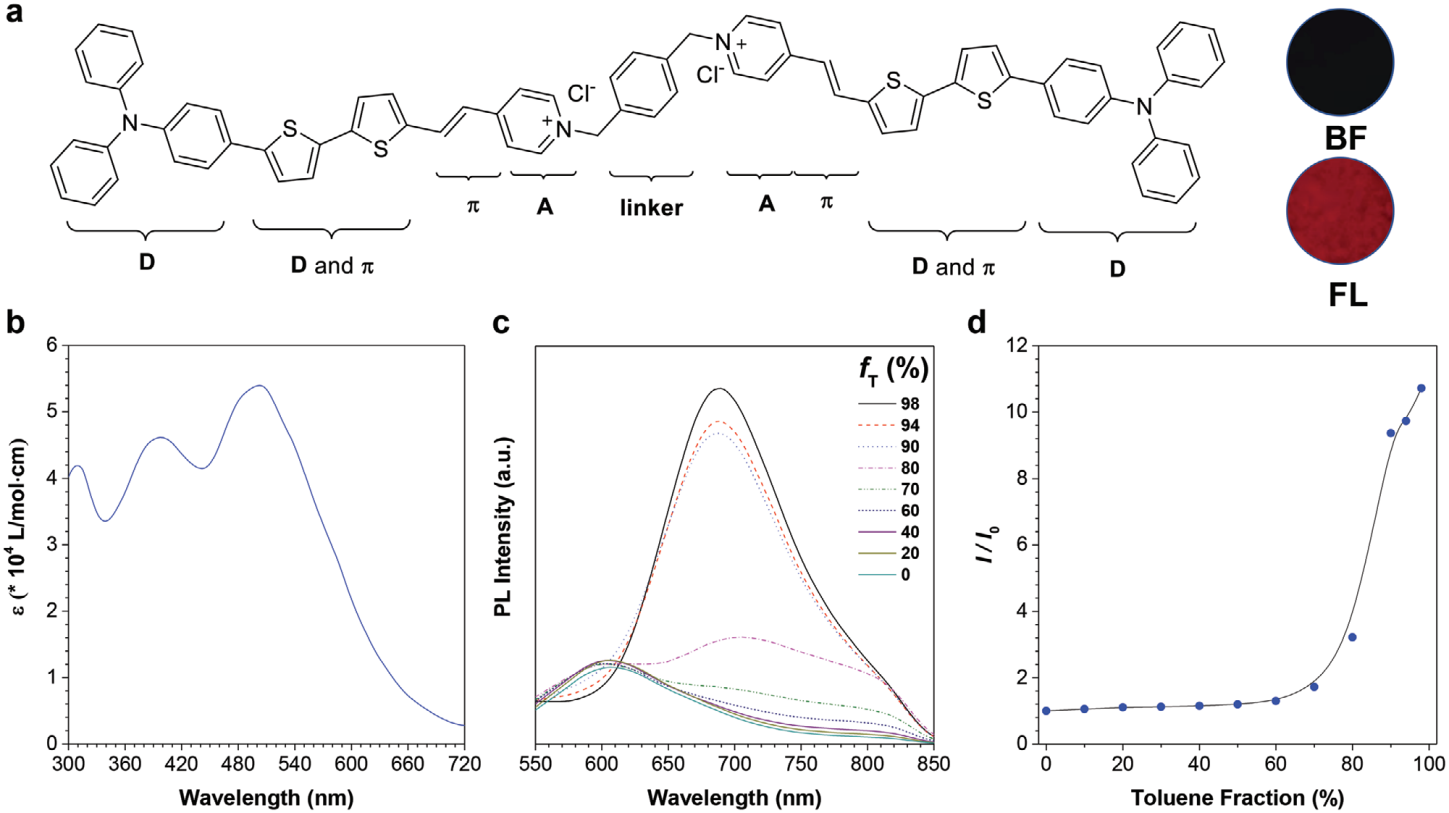
Molecular structure and photophysical properties of DTTPB. a) The chemical structure of DTTPB. Inset: bright‐field (BF) and fluorescence (FL) images of the powder of DTTPB. In FL image, blue‐light excitation (460–490 nm) was employed. b) Molar absorption spectrum of DTTPB in PBS. c) Photoluminescence (PL) spectra of DTTPB (2.5 µm) in the solvent mixtures of DMSO and toluene with different toluene fractions (*f*_T_). Excitation wavelength: 505 nm. d) The plot of the relative FL intensity of DTTPB versus *f*_T_. *I*_0_ and *I* are the PL intensities of DTTPB at 689 nm in DMSO and DMSO/toluene mixtures, respectively.

Human coronaviruses (including COVID‐19) are enveloped viruses with lipid bilayer covering their capsid and genetic materials, which could provide additional protection to the virus outside the host cell.^[^ ^1^, ^2^, ^3^, ^30^ ^]^ Since virus surface proteins vary among different human coronavirus species and mutants, lipid envelope may serve as a conservative target for PSs.^[^ ^31^, ^32^ ^]^ Thus, we designed and synthesized an AIE‐active membrane‐targeting PS with near‐infrared FL, DTTPB (**Figure** **1**a), and employed it for photodynamic combat of human coronaviruses. DTTPB exhibited a broad absorption band covering the whole visible light range and a high molar absorption coefficient. Besides, DTTPB could specifically target plasma membranes in different cell types, as well as the lipid bilayer envelope of virus, such as human coronaviruses (HCoV‐OC43 and HCoV‐229E). Furthermore, DTTPB could also sensitize the generation of ROS upon white‐light irradiation. DTTPB showed higher ROS and ^1^O_2_ sensitizing efficiency than the widely used PS, Rose Bengal (RB), under white‐light irradiation, and were employed for highly efficient PDI of the virus. In the presence of 0.8 µm DTTPB, more than 99.9% human coronaviruses (HCoV‐229E) could be inactivated after irradiation with 9 mW cm^−2^ white light for 20 min.

## 2 Results and Discussion

### 2.1 Synthesis and Photophysical Properties of DTTPB

Our molecular design of the PS was mainly based on three considerations: high ROS sensitizing efficiency, good membrane targeting, and non‐planar conformation for AIE attributes. To construct PS with high ROS sensitizing efficiency, donor‐π‐acceptor (D‐π‐A) structure feature was adopted.^[^ ^33^ ^]^ Such structure feature could lower the energy gap between singlet excited state and triplet excited triplet, which was highly beneficial for facilitating intersystem crossing and could increase ROS sensitizing efficiency.^[^ ^34^ ^]^ In order to target the envelope of the virus, amphiphilic structure was preferred, mimicking the structure of phospholipid of the membrane.^[^ ^35^ ^]^ Based on these considerations, the molecular structure of DTTPB was adopted (Figure 1a), in which triphenylamine (TPA) segment and bithiophene served as the electron donor, the carbon‐carbon double bond functioned as the π‐bridge, and the pyridinium acted as electron acceptor, which constructed a D‐π‐A structure feature. Besides, the donor and π‐bridge of DTTPB were hydrophobic in nature, while the acceptor pyridinium salt with positive charge was hydrophilic, which together would contribute to its targeting to membranes. Furthermore, the non‐planar TPA units and overall bent conformation of DTTPB formed the structural foundation for its AIE attributes. Accordingly, DTTPB was synthesized through a simple condensation reaction of an aldehyde with pyridinium (Scheme S1, Supporting Information) with 74.3% yield. Both intermediate and the final product of DTTPB were characterized by ^1^H NMR and HRMS (Figures S1–S4, Supporting Information), from which satisfactory results corresponding to their molecular structures were obtained.

We first investigated the photophysical properties of DTTPB. DTTPB exhibited a broad absorption band covering the whole visible light range, with an absorption maximum at 505 nm and a high molar absorption coefficient of 5.4 × 10^4^ L mol^−1^ cm^−1^ (Figure 1b). Next, we measured the UV‐Vis absorption spectra of DTTPB in solvent mixtures with different toluene fractions (*f*_T_). As toluene was a bad solvent for DTTPB, increasing *f*_T_ resulted in the reduction of solvating power of the solvent mixture and finally led to the precipitation of DTTPB. As shown in Figure S5, Supporting Information, in the beginning, the maximum wavelength was not changed with the increase in *f*_T_. When *f*_T_ was raised to over 80%, an obvious redshift in the maximum absorption band was observed, indicative of the intermolecular interactions of DTTPB in the aggregated states. The DMSO solution of DTTPB fluoresced weakly with a red emission band peaked 606 nm (Figure 1c) and a large Stokes shift of over 100 nm. In DMSO/toluene mixtures with *f*_T_ lower than 60%, DTTPB exhibited faint FL. Further increasing *f*_T_ to beyond 70% led to aggregate formation and resulted in a quick FL enhancement with a bathochromic shift from 606 to 689 nm, which could be ascribed to the intermolecular interactions between DTTPB molecules in the aggregates formed in DMSO/toluene solvent mixtures with high *f*_T_. The FL intensity of DTTPB in 98% toluene solution was 10.7‐folds higher than that in pure DMSO (Figure 1d), affirmatively demonstrating the typical AIE property of DTTPB. The FL spectra of DTTPB extended to over 850 nm, which fell into the near‐infrared range.

### 2.2 Fluorescence Imaging of Plasma Membrane with DTTPB

**Figure 2:**
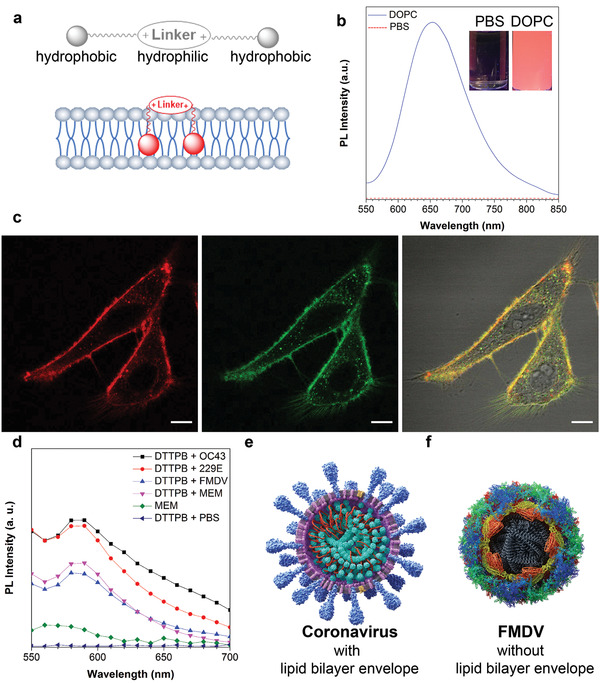
a) Schematic illustration of DTTPB structural characteristics and its interaction with membrane structure. b) PL spectra of DTTPB (5 µm) in PBS solution with or without 5 mg mL^−1^ DOPC. Inset: corresponding FL images of DTTPB in PBS solution with or without DOPC under a hand‐held UV lamp at 365 nm. c) Co‐localization imaging of HeLa cells stained with DTTPB (red) and CellMask Green (green). The 561 nm laser and 620–720 nm emission filter were used for DTTPB. The 488 nm laser and 510–550 nm emission filters were used for CellMask Green. Scale bar: 10 µm. d) PL spectra of DTTPB (5 µm) in different kinds of virus solutions, MEM media, or PBS solution. Excitation wavelength: 490 nm. e,f) Schematic illustrations of the structural characteristics of (e) human coronaviruses and (f) FMDV.

**Figure****2**a illustrates the design principle of DTTPB for membrane targeting. The TPA, bithiophene, and carbon‐carbon double bond units are hydrophobic in nature, which would be embedded into the “non‐polar tail” of phospholipid through hydrophobic interactions.^[^ ^36^ ^]^ The pyridinium salts are positively charged hydrophilic units, which align on the surface of lipid bilayers and bind to the negatively charged “polar head” of phospholipid through strong electrostatic interactions.^[^ ^37^ ^]^ Two amphiphilic chromophores are linked together through a phenyl group. Furthermore, upon binding to membrane structures, the emitting moieties of DTTPB are embedded into the phospholipids. Such binding event restricts the intramolecular motion of DTTPB and gives rise to its FL, which is typical of the AIE phenomenon.^[^ ^38^, ^39^ ^]^

To demonstrate the membrane specificity of DTTPB, a phospholipid, 1,2‐dioleoyl‐sn‐glycero‐3‐phosphocholine (DOPC), was used to investigate the interaction between DTTPB and phospholipid. DTTPB showed almost no FL in PBS solution (Figure 2b) and the addition of 5 mg mL^−1^ DOPC to PBS could remarkably enhance its FL peak at 653 nm with 1832‐fold increase in FL intensity. Such drastic change in FL could be clearly visualized by naked eyes under irradiation with a 365 nm hand‐held UV lamp (Figure 2b). Compared with the FL spectrum of DTTPB in aggregated state (98% toluene), the FL peak of DTTPB in DOPC‐containing PBS solution was blue‐shifted by over 30 nm (from 689 to 653 nm), which might be originated from the different interactions involved in these two states.

As the phospholipids of the virus envelope are originated from the host cells during virus release from host cells, either through lysis or budding, the species and ratio of phospholipids in virus membrane should be similar to those of its host cells.^[^ ^40^ ^]^ We then examined the subcellular localization of DTTPB in living mammalian cell lines. Human cervical carcinoma cells (HeLa), human liver carcinoma cells (HepG2), and human embryonic kidney cells (HEK‐293T) were incubated with 5 µm DTTPB for 60 min, followed by the collection of FL images by using a confocal laser scanning microscopy. In HeLa cells, DTTPB specifically targeted cell surface regions and endowed HeLa cell membrane with red FL. The FL signal from DTTPB overlapped with that from the commercial plasma membrane probe, CellMask Green (Figure 2c and Figure S6, Supporting Information). The Pearson's correlation coefficient was determined to be 0.87, which suggested its specificity to the plasma membrane. DTTPB also demonstrated high selectivity for the plasma membrane of the other two tested cell lines of HEK‐293T and HepG2 (Figure S7, Supporting Information). These results unambiguously proved the membrane specificity of DTTPB, and implicated that DTTPB could bind to the membrane of virus.

Subsequently, we investigated the interactions between DTTPB and virus. DTTPB were incubated with three kinds of viruses in minimum essential medium (MEM), HCoV‐OC43, HCoV‐229E, and foot‐and‐mouth disease virus (FMDV), and the FL spectra of these solutions were collected. Besides, the FL spectra of DTTPB in PBS and MEM, and MEM alone were also recorded for comparison. As illustrated in Figure 2d, DTTPB emitted faintly in PBS. MEM could slightly turn on the FL of DTTPB, presumably due to the electrostatic interaction of DTTPB with the components in MEM. The FL intensity of DTTPB could be increased by the presence of human coronavirus (HCoV‐OC43 and HCoV‐229E) rather than FMDV, which suggested the selective binding of DTTPB towards human coronaviruses. The FL intensity of DTTPB incubated with HCoV‐OC43 or HCoV‐229E was about 1.5‐fold higher than that without virus. The different FL responses between human coronaviruses and FMDV could be ascribed to their distinctly different structures: human coronaviruses were enveloped viruses with phospholipid bilayers covering their capsid (Figure 2e), while FMDV was non‐enveloped picornavirus (Figure 2f).^[^ ^30^, ^41^ ^]^ DTTPB could bind to the envelope of human coronavirus through interacting with its phospholipids. Upon binding to human coronavirus, the FL of DTTPB was peaked at 590 nm, which was blue‐shifted as compared to that in the presence of DOPC. The hypsochromic shift should be stemmed from the additional components in the virus membrane, such as other phospholipids and membrane proteins, which influenced the binding and conformation of DTTPB. The results presented above clearly proved the binding of DTTPB towards the membrane structure of coronaviruses, which formed the foundation for PDI.

### 2.3 Cytotoxicity of DTTPB

For a PS to be employed for PDI of virus on a high‐touch surface, it should exert a minimum effect on humans. We thus evaluated the cytotoxicity of DTTPB by employing the cell counting kit‐8 (CCK‐8) method. Mammalian cell line, mouse embryonic fibroblast cells (NIH‐3T3), and two host cell lines, baby hamster kidney fibroblast cells (BHK‐21 for FMDV) and human fetal lung fibroblast cells (MRC‐5 for HCoV‐OC43 and HCoV‐229E), were incubated with different concentrations of DTTPB, ranging from 1.0 to 15.0 µm. As illustrated in Figure S8, Supporting Information, the cells were still highly viable even after incubation with 15 µm DTTPB for 24 h, which demonstrated the excellent biocompatibility of DTTPB.

### 2.4 Evaluating the ROS Sensitizing Efficiency of DTTPB

**Figure 3:**
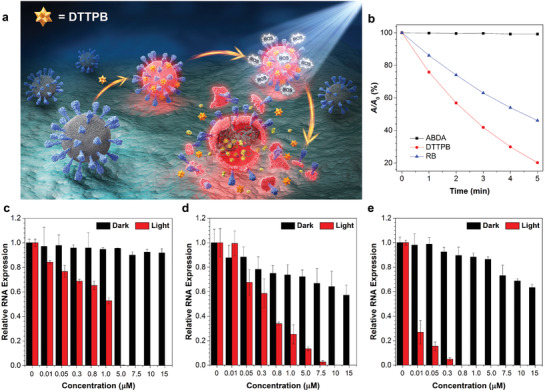
a) Schematic illustration of the PDI process of coronaviruses with DTTPB. b) Decomposition rates of ABDA in the absence or presence of DTTPB or RB under light irradiation (20 mW cm^−2^), where *A*_0_ and *A* are the initial and final absorbance of ABDA at 378 nm, respectively. c–e) qPCR studies of relative RNA expression of (c) FMDV, (d) HCoV‐OC43, and (e) HCoV‐229E. The viruses were treated with white‐light irradiation (9 mW cm^−2^) for 20 min, followed by infecting host cells and RNA extraction. Data were expressed as mean ± SE, number of duplicates: 3.

The membrane targeting of DTTPB, and strong and broad absorption of DTTPB covering the whole visible light region are advantageous for anchoring to and PDI of coronavirus. As illustrated in **Figure** **3**a, upon incubation with coronavirus, DTTPB would bind to the envelope of coronavirus, and its FL could be lit up. Besides, upon white‐light irradiation, ROS will be generated, which would destroy the structures of biomolecules of the virus and lead to its inactivation.

A key factor determining PDI efficiency was its ability to sensitize ROS generation. In our design of the PS, D‐π‐A structure feature was included to increase its ROS sensitizing efficiency through facilitating intersystem crossing. The ROS generation efficiency of DTTPB was first evaluated by the use of 2′,7′‐dichlorodihydrofluorescein diacetate (H2DCF‐DA) as an indicator. H2DCF‐DA emitted faintly, and reaction with ROS would turn on its FL at around 534 nm. Thus, by recording the FL of H2DCF‐DA, the efficiency of ROS production could be determined. As depicted in Figure S9, Supporting Information, DTTPB or H2DCF‐DA alone was non‐emissive or weakly emissive upon irradiation with white light (20 mW cm^−2^) for up to 160 s, suggesting no or low ROS produced. In contrast, in the presence of both DTTPB and H2DCF‐DA, the FL intensity at 534 nm increased gradually with prolonged exposure time under white‐light irradiation (20 mW cm^−2^). The FL intensity increased by 220‐fold after 160 s irradiation, obviously proving the high ROS sensitizing efficiency of DTTPB.

^1^O_2_ was considered as the first ROS generated during PDT, we thus evaluated the ^1^O_2_ sensitizing ability of DTTPB by measuring absorption changes of the ^1^O_2_ indicator, 9,10‐anthracenediyl‐bis(methylene)dimalonic acid (ABDA), under white‐light irradiation (20 mW cm^−2^).^[^ ^42^ ^]^ For comparison, the photosensitizing ability of a widely used PS, Rose Bengal (RB), was also evaluated under the same experimental conditions. As illustrated in Figure S10, Supporting Information, and Figure 3b, the absorbance of ABDA (50 mm) at 378 nm in the solution containing DTTPB (5 µm) gradually decreased to 20.2% of its original level upon white‐light irradiation for 5 min. In the control group without DTTPB, the absorption profile of ABDA solution was not changed under the white‐light irradiation. Under the same experimental conditions, the absorbance of ABDA at 378 nm in a solution containing 5 µm RB only decreased to 46.1% of its original value. These results clearly demonstrated that DTTPB could efficiently sensitize the generation of ^1^O_2_ for PDI applications. Encouragingly, the photosensitizing efficiency of DTTPB under white‐light irradiation was superior to RB, and the decomposition rate constants of ABDA in the presence of DTTPB were 2.06‐folds of that for RB (Figure S11, Supporting Information).

### 2.5 qPCR for Evaluating Viral RNA Copies in Host Cells

DTTPB with broad absorption band, high molar absorbance and ROS generation ability, and excellent membrane‐targeting ability of DTTPB was the ideal candidate for photodynamic antivirus applications. We thus explored its potential for PDI of coronavirus. FMDV, HCoV‐OC43, and HCoV‐229E were incubated with different concentrations of DTTPB for 10 min, followed by treatment with 9 mW cm^−2^ white‐light irradiation or storage in dark for 20 min. Afterwards, the FMDV was used to infect BHK‐21 cells, while the HCoV‐OC43 and HCoV‐229E were employed for infecting MRC‐5 cells. After infection for 24 h, the total RNA from the infected cells was extracted and the level of viral RNA was determined by real‐time quantitative polymerase chain reaction (qPCR). As illustrated in Figure 3c–e, DTTPB exerted low dark toxicity towards the two coronaviruses, but showed no toxicity to FMDV. At a DTTPB concentration of 15 µm, the viral RNA expression levels for FMDV, HCoV‐OC43, and HCoV‐229E in corresponding host cells, were 91.7, 57.1, and 63.4%, respectively. In contrast, white‐light treatment could effectively reduce RNA copies in infected cells, suggesting the effective PDI of the virus. As shown in Figure 3c, FMDV RNA level dropped to zero for BHK‐21 cells incubated with FMDV treated with 5 µm DTTPB and white‐light irradiation for 20 min. For HCoV‐OC43 and HCoV‐229E, with white‐light irradiation, their RNA copies could not be detected by qPCR at DTTPB concentrations higher than 7.5 (Figure 3d) and 0.5 µm (Figure 3e), respectively. For comparison, viruses treated with white‐light irradiation alone were also used to infect corresponding host cells, and the results indicated that light irradiation alone showed no inactivation effect on all the three species of tested viruses, which ambiguously excluded the possibility that the PDI effect in our experimental group was originated from light irradiation (Figure S12, Supporting Information).

### 2.6 DTTPB Reduced the Expression Level of FMDV 3D Protein

To verify the DTTPB‐mediated PDI of the virus, FMDV 3D protein, a key protein for FMDV replication in host cells was selected as an example, and its expression level was evaluated by western blotting.^[^ ^43^ ^]^ FMDV 3D protein is a virus‐encoded RNA‐dependent RNA polymerase, which serves as the catalytic component in RNA replication and plays important roles in the life cycle of RNA viruses.^[^ ^44^ ^]^ Identification of FMDV 3D protein in host cells is thus regarded as a sign of virus infection. In our experiments, FMDV was first treated with varying concentrations of DTTPB and 20 min white‐light irradiation, followed by incubation with BHK‐21 cells for 24 h at an FMDV concentration of 2.5 × 10^−4^ PFU/cell. Afterwards, equal numbers of cells were harvested and lysed, followed by analysis with western blotting for FMDV 3D protein. As shown in Figure S13, Supporting Information, for virus without PDI treatment, FMDV 3D protein could be detected in its host cells, demonstrating their successful invasion into and amplification inside host cells. For FMDV treated with varying concentrations of DTTPB and white‐light irradiation for 20 min, the FMDV 3D protein could not be detected in the host cells after incubation. The results clearly proved the effective inactivation of FMDV, which lost invading ability after DTTPB‐mediated PDI.

### 2.7 DTTPB‐Mediated PDI Decreased the Expression Level of HCoV‐OC43 Protein in Host Cells

**Figure 4:**
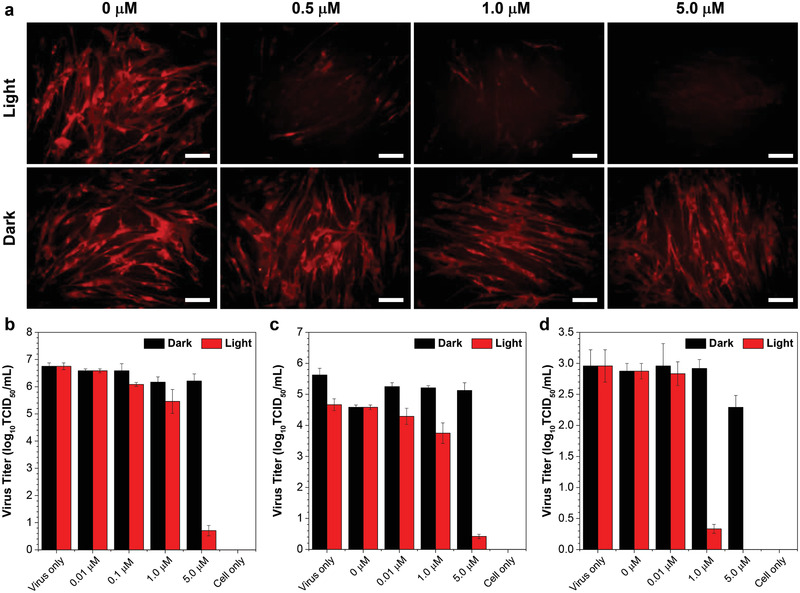
a) Immunofluorescence studies of MRC‐5 cells infected with HCoV‐OC43, which were pre‐treated with different concentrations of DTTPB and then irradiated with 9 mW cm^−2^ white light for 20 min. These MRC‐5 cells were then subjected to immunostaining with primary anti‐OC43 specific polyclonal antibody and TRITC Goat Anti‐Mouse IgG (H+L) secondary antibody, followed by imaging with a FL microscope (λ_ex_: 510–550 nm, λ_ex_: 570–750 nm). Scale bar: 50 µm. b–d) TCID_50_ assay for detecting the live viral (b) FMDV, (c) HCoV‐OC43, (d) and HCoV‐229E titers. The viruses were incubated with different concentrations of DTTPB with or without 9 mW cm^−2^ white‐light irradiation for 20 min. Data were expressed as mean ± SE, number of duplicates: 8.

To provide an intuitive view of the PDI results, immunofluorescence assay was used to evaluate the expression level of virus protein in infected cells, by employing commercially available antibodies against HCoV‐OC43 as an example. In this experiment, HCoV‐OC43 were treated with varying concentrations of DTTPB for 10 min, followed by exposure to white light or storage in dark for 20 min. Afterwards, MRC‐5 cells were infected with these HCoV‐OC43 and then incubated for an additional 24 h. These MRC‐5 cells were then subjected to immunostaining with primary anti‐OC43 specific polyclonal antibody and TRITC Goat Anti‐Mouse IgG (H+L) secondary antibody, followed by imaging with an FL microscope. The virus antigen levels in each group were analyzed by immunofluorescence analysis with Image J. As shown in **Figure** **4**a, the red FL from TRITC in MRC‐5 cells decreased gradually with increasing concentrations of DTTPB employed for PDI of HCoV‐OC43, which suggested the decreased antigen levels at high DTTPB concentrations. At a DTTPB concentration of 5 µm, almost no red FL from TRITC was observed, which implied the low antigens levels inside these MRC‐5 cells. In the control groups where HCoV‐OC43 was not irradiated, MRC‐5 cells showed strong red FL at all the tested concentrations, which demonstrated the necessity for involving light for virus inactivation. These FL images were further processed with Image J, and the mean gray values of individual cells were collected. As shown in Figure S14, Supporting Information, the average gray value was 17.9, 9.8, and 3.901 per cell at different concentrations of DTTPB with white‐light irradiation, respectively, which were much lower than the positive control (virus only, 40.1). However, without white‐light irradiation, the average FL was 34.1, 32.9, and 25.5 at the same concentration of DTTPB, respectively. These results clearly demonstrated that DTTPB can effectively prevent the infection and the spread of HCoV‐OC43 through PDT.

### 2.8 Viral Titering‐Median Tissue Culture Infectious Dose (TCID_50_) Assay

In the next place, TCID_50_ assay was used to evaluate the number of infectious virus particles. In TCID_50_ assay, the viruses were incubated with different concentrations of DTTPB and then exposed to white light for 20 min, followed by incubation with corresponding host cells for 3 days and quantification of the plaques formed. As shown in Figure 4b–d, with 5 µm of DTTPB and light treatment, the log_10_TCID_50_ of FMDV, HCoV‐OC43, and HCoV‐229E were significantly decreased by nearly 89.5 (Figure 4b), 92 (Figure 4c), and 100% (Figure 4d), as in sharp contrast with the control group (virus only). In contrast, the titers of FMDV, HCoV‐OC43, and HCoV‐229E stored in dark were just decreased slightly.

We also investigated the morphology changes of BHK‐21 cell by using an inverted microscope (Figure S15, Supporting Information). Without FMDV infection (cell only), BHK‐21 cells were in healthy state with uniform spindle shape, and were polarity arranged without any cytopathic effects (CPE). After 24 h post‐infection with virus, CPE was observed in the positive control (virus only) group and cells treated with white light alone (0 µm). By increasing the concentration of DTTPB from 0.01 to 10 µm, the CPE decreased gradually. It is noteworthy that all the BHK‐21 cells displayed almost no CPE after 24 h post infection by FMDV treated with 10 µm DTTPB and white‐light irradiation, which indicated that DTTPB could effectively inactivate FMDV and thus provide strong protection for BHK‐21 cells.

### 2.9 PDI of Viruses on High‐Touch Surface

**Figure 5:**
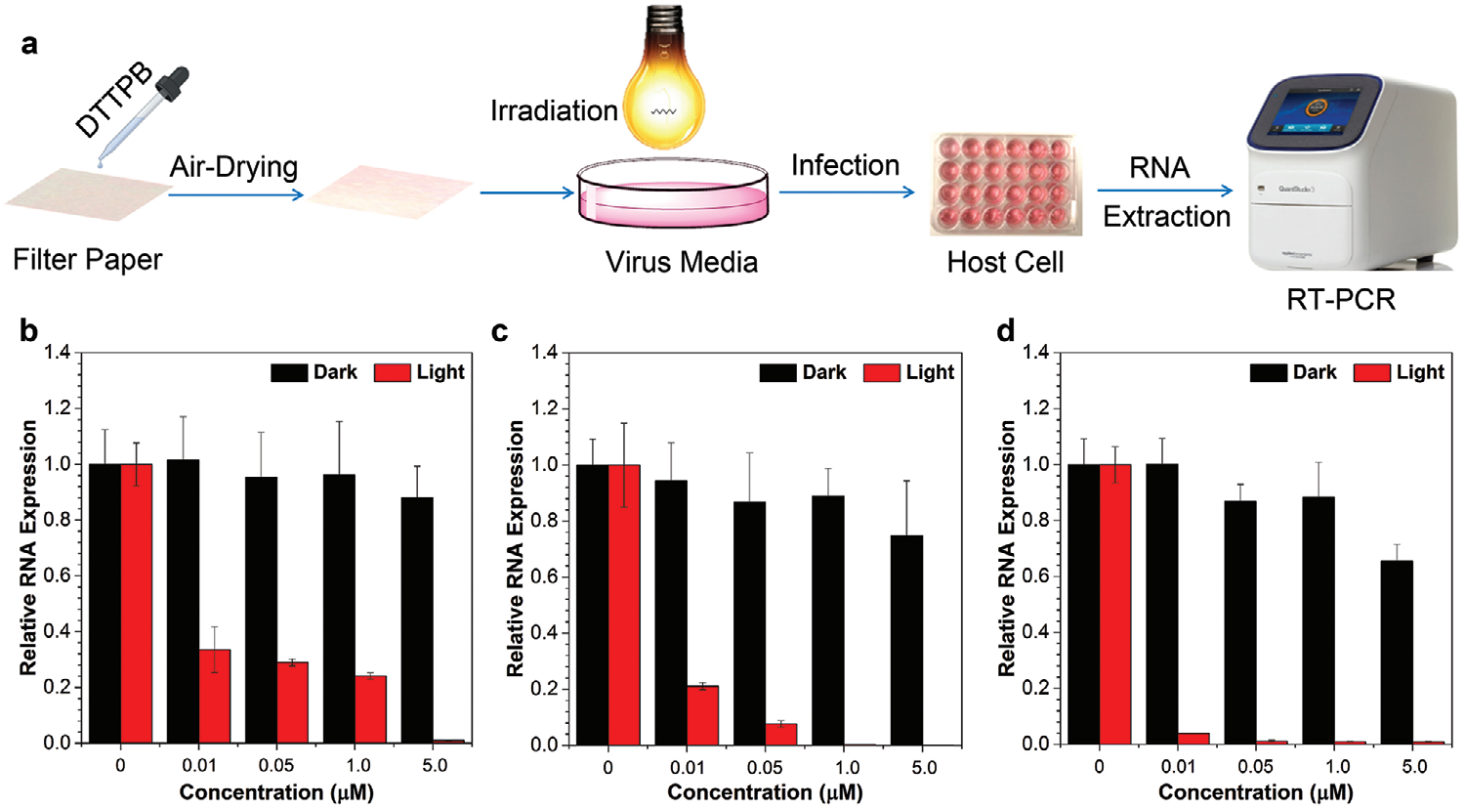
Investigation on the PDI effect of DTTPB on simulated high‐touch surfaces. a) Schematic illustration of experimental procedures for DTTPB‐mediated PDI of virus. b–d) qPCR analyses of PDI effect of DTTPB‐mediated PDI on (b) FMDV, (c) HCoV‐OC43, and (d) HCoV‐229E. In this experiment, varying concentrations of DTTPB were sprayed onto filter papers, and placed in a 24‐well plate and incubated with FMDV, HCoV‐OC43, or HCoV‐229E for 10 min, and then treated with or without white‐light irradiation (9 mW cm^−2^) for 20 min. These viruses were collected and employed for incubating with corresponding host cells for 24 h, followed by qPCR analysis. Data were expressed as mean ± SE, number of duplicates: 3.

We further tested the feasibility of applying DTTPB in PDI of virus on high‐touch surfaces. In this experiment, filter paper was employed for simulating the high‐touch surface and the experimental procedures are illustrated in **Figure** **5**a. Ethanol solutions containing different concentrations of DTTPB were dropped onto sterilized filter paper. After drying under air, the filter paper was immersed in virus‐containing media, and then treated with or without white‐light irradiation. Subsequently, the BHK‐21 or MRC‐5 cells were incubated with the corresponding virus‐containing media for 24 h, followed by evaluating the level of viral RNA in the infected cells with qPCR. As shown in Figure 5b–d and Figure S16, Supporting Information, DTTPB could still efficiently inactivate all the three kinds of virus on filter paper with white‐light irradiation. At a DTTPB concentration of 0.01 µm, the relative RNA expression levels were decreased to 33.5, 21.0, and 3.9% for FMDV (Figure 5b), HCoV‐OC43 (Figure 5c), and HCoV‐229E (Figure 5d), respectively. When DTTPB concentration was increased to 1.0 µm, the relative RNA expression levels were decreased to 24, 0.4, and 0.9%, respectively, which ambiguously demonstrated that DTTPB could effectively inactivate viruses in environment and proved its great potential in photodynamic combat of SARS‐CoV‐2. The PDI performance of DTTPB on simulated high‐touch surface was better than that in culture media. This may be ascribed to the further restriction of intramolecular motions by absorbing onto high‐touch surfaces, which could enhance ROS sensitizing efficiency of DTTPB. The results demonstrated the potential of employing DTTPB to prevent the spread of infectious pathogens in public places.

## 3 Conclusion

To sum up, we've developed a near‐infrared membrane‐targeting PS, DTTPB, for photodynamic combat of virus. DTTPB showed typical AIE property with broad absorption band and high molar absorbance covering the whole visible light region. Besides, it also demonstrated excellent ROS sensitizing ability, with higher ^1^O_2_ sensitizing efficiency than the widely used PS, RB. DTTPB could selectively bind to and light up the cell membrane of mammalian cells, suggestive of its targeting to virus envelope. It exhibited good performance in the PDI of all the three tested viruses, as evaluated by the decreased expression of virus RNA copies after incubating with host cells. Specially, it exhibited highly efficient antiviral performance for the human coronavirus, HCoV‐229E: at a low concentration of 0.8 µm DTTPB and with white‐light irradiation, almost no HCoV‐229E RNA copies could be detected in host cells after incubation for 24 h. Similar conclusions could be drawn from western blotting analysis and immunofluorescence studies. The DTTPB‐mediated PDI of virus was also supported by viral tittering and examination of CPE, and the results clearly proved that DTTPB could efficiently prevent the infection and the spread of virus, and therefore directly provide strong protection for host cells. Furthermore, DTTPB also exhibited excellent performance in simulated high‐tough surfaces, demonstrative of its promising practical applications. As far as we are concerned, this contribution represents the first report of employing membrane‐targeting AIE‐type PS for efficient photodynamic killing of human coronaviruses. This work is expected to inspire new preventive strategies for combating with COVID‐19 and controlling the pandemic situation.

## Conflict of Interest

The authors declare no conflict of interest.

## Supporting information

Supporting Information

## Data Availability

Data available on request from the authors.

## References

[1] P. Zhou, X.‐L. Yang, X.‐G. Wang, B. Hu, L. Zhang, W. Zhang, H.‐R. Si, Y. Zhu, B. Li, C.‐L. Huang, H.‐D. Chen, J. Chen, Y. Luo, H. Guo, R.‐D. Jiang, M.‐Q. Liu, Y. Chen, X.‐R. Shen, X. Wang, X.‐S. Zheng, K. Z, Q.‐J. Chen, F. Deng, L.‐L. Liu, B. Yan, F.‐X. Zhan, Y.‐Y. Wang, G.‐F. Xiao, Z.‐L. Shi, Nature 2020, 579, 270. 32015507 10.1038/s41586-020-2012-7 PMC7095418

[2] F. Wu, S. Zhao, B. Yu, Y.‐M. Chen, W. Wang, Z.‐G. Song, Y. Hu, Z.‐W. Tao, J.‐H. Tian, Y.‐Y. Pei, M.‐L. Yuan, Y.‐L. Zhang, F.‐H. Dai, Y. Liu, Q.‐M. Wang, J.‐J. Zheng, L. Xu, E. C. Holmes, Y.‐Z. Zhang, Nature 2020, 579, 265. 32015508 10.1038/s41586-020-2008-3 PMC7094943

[3] Coronaviridae Study Group of the International Committee on Taxonomy of Viruses, Nat. Microbiol. 2020, 5, 536.32123347

[4] S. Gago, S. F. Elena, R. Flores, R. Sanjuán, Science 2009, 323, 1308. 19265013 10.1126/science.1169202

[5] P. V'kovski, A. Kratzel, S. Steiner, H. Stalder, V. Thiel, Nat. Rev. Microbiol. 2021, 19, 155. 33116300 10.1038/s41579-020-00468-6 PMC7592455

[6] S. Su, L. Du, S. Jiang, Nat. Rev. Microbiol. 2021, 19, 211. 33067570 10.1038/s41579-020-00462-y PMC7566580

[7] B. Vellingiri, K. Jayaramayya, M. Iyer, A. Narayanasamy, V. Govindasamy, B. Giridharan, S. Ganesan, A. Venugopal, D. Venkatesan, H. Ganesan, K. Rajagopalan, P. K. S. M. Rahman, S.‐G. Cho, N. S. Kumar, M. D. Subramaniam, Sci. Total Environ. 2020, 725, 138277. 32278175 10.1016/j.scitotenv.2020.138277 PMC7128376

[8] A. Widders, A. Broom, J. Broom, Infect. Dis. Health 2020, 25, 210. 32473952 10.1016/j.idh.2020.05.002 PMC7237903

[9] W. A. Rutala, D. J. Weber, *Guideline for Disinfection and Sterilization in Healthcare Facilities*, Centers for Disease Control and Prevention: Atlanta, GA 2008, pp. 1−163, https://www.cdc.gov/infectioncontrol/guidelines/disinfection/index.html.

[10] L. Dietz, P. F. Horve, D. A. Coil, M. Fretz, J. A. Eisen, K. V. D. Wymelenberg, mSystems 2020, 5, e00245. 32371473 10.1128/mSystems.00375-20 PMC7205520

[11] S. M. Imani, L. Ladouceur, T. Marshall, R. Maclachlan, L. Soleymani, T. F. Didar, ACS Nano 2020, 14, 12341. 33034443 10.1021/acsnano.0c05937

[12] E. Kim, E.‐K. Lim, G. Park, C. Park, J.‐W. Lim, H. Lee, W. Na, M. Yeom, J. Kim, D. Song, S. Haam, Adv. Mater. 2021, 2005927, 10.1002/adma.202005927. 33586180

[13] D. E. Dolmans, D. Fukumura, R. K. Jain, Nat. Rev. Cancer 2003, 3, 380. 12724736 10.1038/nrc1071

[14] V.‐N. Nguyen, Y. Yan, J. Zhao, J. Yoon, Acc. Chem. Res. 2021, 54, 207. 33289536 10.1021/acs.accounts.0c00606

[15] B. Yang, Y. Chen, J. Shi, Chem. Rev. 2019, 119, 4881. 30973011 10.1021/acs.chemrev.8b00626

[16] F. Cieplik, D. Deng, W. Crielaard, W. Buchalla, E. Hellwig, A. Ahmad, T. Maisch, Crit. Rev. Microbiol. 2018, 44, 571. 29749263 10.1080/1040841X.2018.1467876

[17] A. Kamkaew, S. H. Lim, H. B. Lee, L. V. Kiew, L. Y. Chung, K. Burgess, Chem. Soc. Rev. 2013, 42, 77. 23014776 10.1039/c2cs35216h PMC3514588

[18] L. Sobotta, P. Skupin‐Mrugalska, J. Mielcarek, T. Goslinski, J. Balzarini, Mini‐Rev. Med. Chem. 2015, 15, 503. 25877599 10.2174/1389557515666150415151505

[19] S. S. Lucky, K. C. Soo, Y. Zhang, Chem. Rev. 2015, 115, 1990. 25602130 10.1021/cr5004198

[20] E. W. Schultz, A. P. Krueger, Exp. Biol. Med. 1928, 26, 100.

[21] X. He, L.‐H. Xiong, Z. Zhao, Z. Wang, L. Luo, J. W. Y. Lam, R. T. K. Kwok, B. Z. Tang, Theranostics 2019, 9, 3223. 31244951 10.7150/thno.31844 PMC6567968

[22] A. Wiehe, J. M. O'Brien, M. O. Senge, Photochem. Photobiol. Sci. 2019, 18, 2565. 31397467 10.1039/c9pp00211a

[23] N. Kipshidze, N. Yeo, N. Kipshidze, Nat. Photonics 2020, 14, 651.

[24] F. Hu, S. Xu, B. Liu, Adv. Mater. 2018, 30, 1801350. 10.1002/adma.201801350 30066341

[25] W. M. Sharman, C. M. Allen, J. E. van Lier, Methods Enzymol. 2000, 319, 376. 10907528 10.1016/s0076-6879(00)19037-8

[26] N. Song, Z. Zhang, P. Liu, Y.‐W. Yang, L. Wang, D. Wang, B. Z. Tang, Adv. Mater. 2020, 32, 2004208. 10.1002/adma.202004208 33150632

[27] Z. Liu, G. Zhang, D. Zhang, Acc. Chem. Res. 2018, 51, 1422. 29771491 10.1021/acs.accounts.8b00069

[28] Y. Wang, L. Feng, S. Wang, Adv. Funct. Mater. 2019, 29, 1806818.

[29] H. Bai, W. He, J. H. C. Chau, Z. Zheng, R. T. K. Kwok, J. W. Y. Lam, B. Z. Tang, Biomaterials 2021, 268, 120598. 33321291 10.1016/j.biomaterials.2020.120598

[30] P. Zimmermann, N. Curtis, Pediatr. Infect. Dis. J. 2020, 39, 355. 32310621 10.1097/INF.0000000000002660 PMC7158880

[31] F. Käsermann, C. Kempf, Antiviral Res. 1997, 34, 65. 9107386 10.1016/s0166-3542(96)01207-7

[32] M. Lorizate, H.‐G. Kräusslich, Biol 2011, 3, a004820. 10.1101/cshperspect.a004820 PMC3179339 21628428

[33] D. Wang, M. M. S. Lee, G. Shan, R. T. K. Kwok, J. W. Y. Lam, H. Su, Y. Cai, B. Z. Tang, Adv. Mater. 2018, 30, 1802105. 10.1002/adma.201802105 30133835

[34] M. Kang, C. Zhou, S. Wu, B. Yu, Z. Zhang, N. Song, M. M. S. Lee, W. Xu, F.‐J. Xu, D. Wang, L. Wang, B. Z. Tang, J. Am. Chem. Soc. 2019, 141, 16781. 31553608 10.1021/jacs.9b07162

[35] L. Shi, Y.‐H. Liu, K. Li, A. Sharma, K.‐K. Yu, M. S. Ji, L.‐L. Li, Q. Zhou, H. Zhang, J. S. Kim, X.‐Q. Yu, Angew. Chem., Int. Ed. 2020, 59, 9962. 10.1002/anie.201909498 31464051

[36] J. Wang, X. Zhu, J. Zhang, H. Wang, G. Liu, Y. Bu, J. Yu, Y. Tian, H. Zhou, ACS Appl. Mater. Interfaces 2020, 12, 1988. 31771326 10.1021/acsami.9b15577

[37] G. Niu, R. Zhang, Y. Gu, J.i. Wang, C. Ma, R. T. K. Kwok, J. W. Y. Lam, H. H.‐Y. Sung, I. D. Williams, K. S. Wong, X. Yu, B. Z. Tang, Biomaterials 2019, 208, 72. 30999153 10.1016/j.biomaterials.2019.04.002

[38] M.‐Y. Wu, J.‐K. Leung, L. Liu, C. Kam, K. Y. K. Chan, R. A. Li, S. Feng, S. Chen, Angew. Chem., Int. Ed. 2020, 59, 10327. 10.1002/anie.201916718 PMC7318220 32163217

[39] J. Zhang, Q. Wang, Z. Guo, S. Zhang, C. Yan, H. Tian, W.‐H. Zhu, Adv. Funct. Mater. 2019, 29, 1808153.

[40] M. Mazzon, J. Mercer, Cell. Microbiol. 2014, 16, 1493. 25131438 10.1111/cmi.12340 PMC4265854

[41] X. Huang, Y. Li, H. Fang, C. Zheng, Virol. J. 2011, 8, 169. 21492421 10.1186/1743-422X-8-169 PMC3097150

[42] B. A. Lindig, M. A. Rodgers, A. P. Schaap, J. Am. Chem. Soc. 1980, 102, 5590.

[43] R. Kumar, M. Hosamani, B. P. Sreenivasa, A. Kotyal, R. Venkataramanan, Indian J. Virol. 2012, 23, 326. 24293820 10.1007/s13337-012-0098-8 PMC3550801

[44] Y. Gao, S.‐Q. Sun, H.‐C. Guo, Virol. J. 2016, 13, 107. 27334704 10.1186/s12985-016-0561-z PMC4917953

