# Supplementary material for: A Membrane‐Targeting Photosensitizer with Aggregation‐Induced Emission Characteristics for Highly Efficient Photodynamic Combat of Human Coronaviruses
Source: Small. 2021 Jun 30;17(30):2101770. doi: 10.1002/smll.202101770 (PMC8420407; doi:10.1002/smll.202101770)
Supplement: Supplementary file 1 — Supporting Information [file SMLL-17-2101770-s001.pdf]

© 2021 Wiley-VCH GmbH

NANO small MICRO

Supporting Information

for *Small,* DOI: 10.1002/smll.202101770

A Membrane-Targeting Photosensitizer with Aggregation-Induced Emission Characteristics for Highly Efficient Photodynamic Combat of Human Coronaviruses

Ming-Yu Wu, Meijia Gu, Jong-Kai Leung, Xinmei Li, Yuncong Yuan, Chao Shen, Lianrong Wang, Engui Zhao,* and Sijie Chen**

Supporting Information

A Membrane-Targeting Photosensitizer with Aggregation-Induced Emission Characteristics for Highly Efficient Photodynamic Combat of Human Coronaviruses

Ming-Yu Wu, Meijia Gu, Jong-Kai Leung, Xinmei Li, Yuncong Yuan, Chao Shen, Lianrong Wang, Engui Zhao* and Sijie Chen**

Dr. M. Gu and Prof. L. Wang

Key Laboratory of Combinatorial Biosynthesis and Drug Discovery, Ministry of Education, School of Pharmaceutical Sciences, Wuhan University, Wuhan, 430071, Hubei, China.

E-mail: lianrong@whu.edu.cn

Dr. M.-Y. Wu, Mr. J.-K. Leung and Dr. S. Chen

Ming Wai Lau Centre for Reparative Medicine, Karolinska Institutet, Hong Kong, China.

E-mail: sijie.chen@ki.se

Dr. E. Zhao

School of Science, Harbin Institute of Technology, Shenzhen, HIT Campus of University Town, Shenzhen 518055, China.

E-mail: zhaoengui@hit.edu.cn

Ms. X. Li, Mr. Y. Yuan and Prof. C. Shen

College of Life Sciences and China Center for Type Culture Collection, Wuhan University, Wuhan, 430071, Hubei, China

Dr. M.-Y. Wu

School of Life Science and Engineering, Southwest Jiaotong University, Chengdu 610031, China

Table of content

1. Experimental Procedures S3
1.1 Materials and general instruments S3
1.2 Synthesis and characterization of DTTPB S4
1.3 Cell and virus culture S4
1.4 CCK-8 assay for the determination of cell cytotoxicity S5
1.5 Fluorescence imaging S6
1.6 Viral infectivity assay S6
1.7 RNA extraction and qPCR S7
1.8 Immunofluorescence S7
1.9 Western blot analysis S8
1.10 $TCID_{50}$ assays S9
1.11 Photoluminescence measurement S9
1.12 PDI of viruses on filter paper S9
2. NMR and Mass Spectra S11
3. Supplementary Figures S13

1. Experimental Procedures

1.1 Materials and general instruments

All chemical reagents were obtained from J&K Scientific, and used without further purification. CCK-8 kit was purchased from MedChemExpress. Minimum essential medium (MEM) was purchased from Gibco. Phosphate buffered saline (PBS), penicillin and streptomycin were purchased from Invitrogen. Dulbecco's modified Eagle's medium (DMEM), fetal bovine serum (FBS), Trypsin-EDTA and CellMask™ Green were purchased from Thermo Fisher Scientific. The antibody against Coronavirus Antibody OC-43 strain was purchased from Millipore (541-8F). The FMDV 3D protein antibody was homemade. HRP Goat Anti-Mouse IgG (H+L) (ABclonal) was used as the secondary antibody. Antibody against GAPDH was obtained from Proteintech (Wuhan, China). Milli-Q water was supplied by Milli-Q Plus System (Millipore Corporation, United States). All the solvents for optical spectroscopic studies were HPLC or spectroscopic grade.

Thin-layer chromatography analyses were performed on silica gel GF 254. Column chromatography purification was carried out on silica gel (200–300 mesh). NMR spectra were recorded using a Bruker AMX-400. Chemical shifts were given in ppm relative to the internal reference TMS, CD_3OD or DMSO-d_6 as the internal standard. The following abbreviations were used in ^{1}H NMR: s = singlet; d = doublet; t = triplet; q = quartet; m = multiplet. High resolution mass spectra were recorded on a Bruker Daltonics Bio TOF mass spectrometer. Fluorescence spectra were obtained using a Horiba Duetta spectrofluorometer with a 10 mm quartz cuvette. UV-Vis absorption spectra were recorded on a Hitachi PharmaSpec UV-1900 UV-Visible spectrophotometer. Fluorescence images were collected on Olympus IX71 inverted fluorescence microscope or Zeiss LSM 880 confocal laser scanning microscope.

1.2 Synthesis and characterization of DTTPB

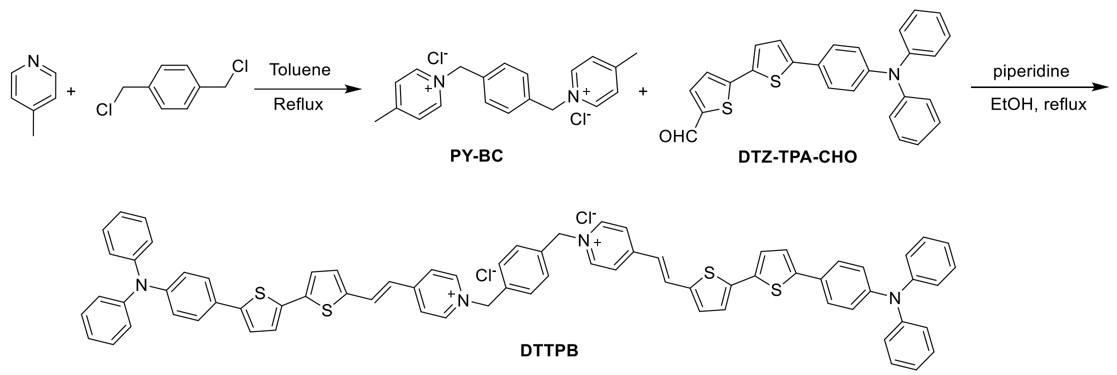

Scheme S1. Synthetic route to DTTPB.

Synthesis of 1-(4-(chloromethyl) benzyl)-4-methylpyridin-1-ium chloride (PY-BC): 4-picoline (4.65 g, 50 mmol) and 1,4-bis(chloromethyl)benzene (4.38 g, 25 mmol) were dissolved in toluene, stirred for 4 h, and thereafter refluxed for 12 h. After cooling and filtrating, the resulting solid was washed with anhydrous ether. 7.4 g white power was obtained with a yield of 82%.^{1}H NMR (400 MHz, CD_3OD) δ: 8.91 (d, J = 6.4 Hz, 2H), 7.97 (d, J = 6.4 Hz, 2H), 7.62 (s, 2H), 5.84 (s, 2H), 2.7 (s, 3H). ^{13}C NMR (100 MHz, CD_3OD) δ: 160.2, 143.1, 134.6, 129.2, 128.3, 63.3, 20.1.

Synthesis of DTTPB: A solution of DTZ-TPA-CHO (96.3 mg, 0.22 mmol)，PY-BC (36.1 mg, 0.1 mmol) and 5 drops of piperidine in dry ethanol (15 mL) was refluxed under nitrogen for 12 h. Afterwards, the reaction mixture was cooled to ambient temperature, followed by filtration and washing with EtOH. DTTPB was obtained as black power in 74.3% yield (89.2 mg). ^{1}H NMR (400 MHz, DMSO-d_6) δ: 9.03 (d, J = 5.2 Hz, 2H), 8.20 (d, J = 6.0 Hz, 2H), 7.61-7.59 (m, 4H), 7.49-7.44 (m, 4H), 7.35 (t, J = 6.0 Hz, 4H), 7.17-7.09 (m, 3H), 7.07 (d, J = 6.8 Hz, 3H), 6.98 (d, J = 6.8 Hz, 2H), 5.74 (s, 2H). HRMS (ESI): m/z $[(M - 2Cl^{-})/2]^{+}$ calculated for $(C_{74}H_{56}N_4S_4)/2$: (1128.3377)/2; found 564.1703.

1.3 Cell and virus culture

HeLa cells, NIH-3T3 cells and HEK-293T cells were cultured in DMEM containing 10% fetal bovine serum (FBS) (Thermo Fisher Scientific) and 1% antibiotic (penicillin and streptomycin) in a 5% CO_2 humidified incubator at 37 ^{o}C.

While HepG2 cells were grown in RPMI 1640 medium (Thermo Fisher Scientific) with 10% fetal bovine serum and 1% antibiotics (penicillin and streptomycin) at 37 °C in a 5% CO_2/95% air incubator. Once the cells reached 80%–90% confluence, they were dissociated into single cells with 0.05% Trypsin-EDTA at 37 °C for 5 min and passaged at a ratio of 1:6–1:19 in a new cell culture dish.

Human embryonic lung fibroblast cells line (MRC-5) and baby hamster kidney cell line (BHK-21) were provided by the China Center for Type Culture Collection. MRC-5 and BHK-21 cells were cultured in MEM medium supplemented with 10% fetal bovine serum, 100 units mL^{-1} penicillin and 100 μg mL^{-1} streptomycin in a 5% CO_2 humidity incubator at 37℃. One day before the infection experiment, the cells were seeded into a 24-well cell culture plate to approximately 70–80% confluence and then infected with virus.

FMDV serotype O viral strains (Akesu/58/2002, GenBank accession no. AF511039) were supplied by the Lanzhou Veterinary Research Institute, Chinese Academy of Agriculture Sciences. Human coronavirus HCoV-229E (ATCC® VR-740) and HCoV-OC43 (ATCC® VR-1558) were purchased from the American Type Culture Collection, MD, USA. Cells were used to propagate viral stocks and measure viral titers in plaque and 50% tissue culture infectious dose ($TCID_{50}$) assays.

The live viruses were incubated with 0.01, 0.05, 0.3, 0.8, 1.0, 5.0, 7.5, 10 and 15 μM of DTTPB. In a typical experiment, 2 μL of a 10 mM stock solution of DTTPB in DMSO were diluted to 1 mL with cell culture medium, followed by further dilution to desired concentration. For the ROS sensitizing, after mixing with the DTTPB, each well of virus was exposed to white light (9 mW/cm^2) for 20 min, another two plates with cells were kept in dark as control.

1.4 CCK-8 assay for the determination of cell cytotoxicity

The cytotoxicity on cells was determined by the standard WST-8 (2-(2-methoxy-4-nitrophenyl)-3-(4-nitrophenyl)-5-(2,4-disulfophenyl)-2H-tetrazolium, monosodium salt) (CCK-8) assay. NIH-3T3, MRC-5 and BHK-21 cells were seeded at a density of

7×10^3 cells per well in 96-well black microplates with 100 μL of culture medium and cultured overnight to reach 70–80% confluence. After that the medium was replaced with 100 μL of fresh medium containing different concentrations of DTTPB (0, 1.0, 2.5, 5, 10, 15 μM), and DMSO was used as a vehicle control. After 24 h of incubation, 10 μL of 12 mM CCK-8 stock solution mixed with 90 μL of phosphate-buffered saline was added to each well for additional 4 h of incubation. The absorbance was measured at 450 nm using the SpectraMax M2 microplate reader (Molecular Devices). Cell viability (%) was calculated as: (OD_{450} sample/OD_{450} control) × 100%.

1.5 Fluorescence imaging

Cells were stained with CellMask™ Green (Thermo Fisher Scientific, 10 min) and 5 μM DTTPB (1h) at 37°C. After the incubation, the cells were washed with DPBS for three times. Confocal imaging was performed using the Zeiss LSM 880 confocal laser scanning microscope equipped with a Plan-Apochromat 63×/1.4 NA oil objective lens, a photo-multiplier tube and a Gallium arsenide phosphide detector driven by the ZEN software (Carl Zeiss). The 561 nm laser and 620–720 nm emission filter were used for DTTPB. The 488 nm laser and 510–550 nm emission filters were used for CellMask™ Green. Digital images were captured and processed by ZEN software (ZEN 2.5 lite) in grayscale and pseudo color.

1.6 Viral infectivity assay

To characterize the effects of DTTPB on virus, cells were infected with FMDV, HCoV-OC43 or HCoV-229E separately, and replication was determined by qPCR later. In brief, an equal number of cells were plated in 24-well plates. When cells reached 70-80% confluence, they were infected with FMDV, HCoV-OC43 or HCoV-229E at a virus concentration of 2.5×10^{-4} PFU/cell. After 24 hours, morphological changes were observed under Olympus IX71 inverted fluorescence microscope.

1.7 RNA extraction and real-time quantitative polymerase chain reaction (qPCR)

After viral infection, cells were collected and RNAs were extracted with TRIzol reagent (Thermo Fisher Scientific) according to the manufacturer protocol. The integrity and purity of RNAs were tested using 1.5% agarose gels, NANO DROP2000 (Thermo), and Agilent 2100. cDNA synthesis was performed according to standard protocols. Briefly, 1 μg RNA was reverse-transcribed using an Oligo(dT)15 Primer (Takara) and M-MLV reverse transcriptase (Promega) with RNase inhibitors (Takara). First, 1 μL Oligo(dT)15 Primer (10 μM) were added into the RNAs with a denaturation step at 70°C for 5 min, followed by mixing evenly with the reverse transcription reaction mixture as follows: 0.5 μL M-MLV reverse transcriptase (Promega), 4 μL 5× reaction buffer, 2 μL dNTP (10 mM), 1 μL RNase inhibitor (Takara), and H_2O to a final volume of 20 μL. The reverse transcription reaction was then performed at 42°C for 60 min and enzyme deactivation at 70°C for 15 min.

Primers of qPCR were designed with Primer Premier 6.0 software (Primer, Canada). The qPCR primers used are listed in Table S1. Each qPCR reaction mixture contained: 5 μL first-strand cDNA from the RT-PCR, 0.25 μL SYBR green dye (Invitrogen), 12.5 μL Premix Taq (Promega), 0.5 μL of each primer (25 μM), and 6.25 μL H_2O to a final volume of 25 μL. The amplification reaction was achieved through one denaturation cycle at 95 °C for 10 min followed by 40 cycles at 94 °C for 30 s, 58 °C for 30 s and a final extension cycle at 72 °C for 20 s in the CFX96TM RT-PCR detection system (BIO-RAD). GAPDH was chosen as a reference gene to normalize the gene expression levels, and results were analyzed with the $2^{-\Delta\Delta Ct}$ method.

Each sample was subjected to three independent replicates. All assays were performed with three independent biological replicates. Data analyses were performed using Rotor-Gene 4.6.

1.8 Immunofluorescence

Cells grown in 24-well plates were washed twice with PBS and fixed with 4%

paraformaldehyde for 30 min followed by permeabilization with 0.5% Triton-X 100 for 15 min. After washing twice with PBS, 5 min each, at room temperature, cells were blocked with 5% bovine serum albumin for 30 min. The cells were then incubated with the primary anti-OC43 specific polyclonal antibody (1:100) overnight at 4°C. Then the cells were washed with PBS for three times, 5 min each, and incubated with TRITC Goat Anti-Mouse IgG (H+L) secondary antibody (1:100) (ABclonal) for 2 h at room temperature. Finally, cells were washed three times with PBS and observed with an Olympus IX71 inverted fluorescence microscope coupled with cellSens software and cell count. For each specimen, ten non-overlapping fields were randomly selected. Averages of these counting results were used for further analysis. Then, the fluorescence intensity of each cell was computed by averaging the value of all the pixels belonging to a certain cell (mean intensity profile, ImageJ software), expressed as pixel gray-scale values. The mean fluorescence was determined for the stained cells (**Figure 4**) and the fluorescence intensity was plotted in **Figure S13**.

1.9 Western blot analysis

Cells underwent different treatments, were washed twice with PBS and proteins were extracted with 2 × SDS buffer and then boiled for 5 min. After centrifuging for 10 min at 10,000 × g, the supernatant was subjected to a 10% SDS-PAGE gel for electrophoresis followed by electrotransfered onto a polyvinylidene fluoride (PVDF) membrane (Bio-Rad) for Western blotting. Prior to the detection of target proteins with specific protein antibody, the transferred membranes were blocked with 5% skim milk (BD Biosciences) for 1 h at room temperature to reduce non-specific binding. Excess milk was washed away three times, and immunoblotting was performed with FMDV 3D or GAPDH protein antibody (1:2000) followed by HRP-conjugated AffiniPure secondary antibody (1:10,000) to develop the blotting results. After washing three times with TBST (containing 0.15 % Tween-20), membrane-bound antibodies were detected using Immobilon Western Chemiluminescent HRP Substrate

(Millipore). Results were presented using the GelDoc XR System (Bio-Rad), and all experiments were repeated independently three times.

1.10 $TCID_{50}$ assays

MRC-5 cells were incubated in 96-well plates at a concentration of 5000 cells/well, and the cells could be used for $TCID_{50}$ when the confluence reached over 70%. And, the virus stock solution was diluted with a 10-fold gradient. The dilution factor for FMDV, HCoV-OC43, and HCoV-229E, the dilution factor was from 10^1 to 10^4, from 10^1 to 10^4, and from 10^1 to 10^3, respectively. Afterwards, the supernatants in the 96-well plate were discarded, and 100 μL per well of virus diluents were added to the 96-well plate. For each dilution, 8 parallel wells were prepared. MEM medium was used as the negative control. Then the infection of virus was performed under 5% CO_2 at 37°C. Cytopathic effect of MRC-5 cells was observed after adding diluted virus to 96-well plates for 3 days, and the numbers of CPE positive wells at each dilution were counted. The titers of virus were calculated according to Reed-Muench's calculation method.

1.11 Photoluminescence measurement

DTTPB (5 mM DMSO stock solution) was added into medium with or without viruses. The mixtures were incubated at room temperature for 20 min (final DTTPB concentration: 5 μM). The mixtures were then added into Corning® Costar 96-Well Black-Bottom Plates (100 μL/well). Photoluminescence (PL) spectra of the mixtures were recorded with Molecular Devices SpectraMax i3x Multi-ModeMicroplate Detection System.

1.12 PDI of viruses on filter paper

Sterilized filter papers were cut into small pieces with sizes of 1 cm × 1 cm, and then randomly divided into control groups and experimental groups with three independent replicates. 10 μL of ethanol containing different concentrations of DTTPB was added

to filter paper with completely immerse. After air-drying for more than 12 h, the filter papers were placed in 24-well plate and incubated with FMDV, HCoV-OC43 or HCoV-229E for 10 min, and then treated with or without white-light irradiation (9 mW/cm^2) for 20 min. The BHK-21 or MRC-5 cells were infected with FMDV, HCoV-OC43 or HCoV-229E separately for 24 h. The replications were then determined by qPCR. In brief, an equal number of cells were plated in 24-well plates. When cells reached 70-80% confluence, they were infected with FMDV, HCoV-OC43 or HCoV-229E. After 24 hours, cells were collected and RNA was extracted with TRIzol reagent (Takara) according to the manufacturer protocol, followed by qPCR analysis of RNA copies.

2. NMR and Mass Spectra

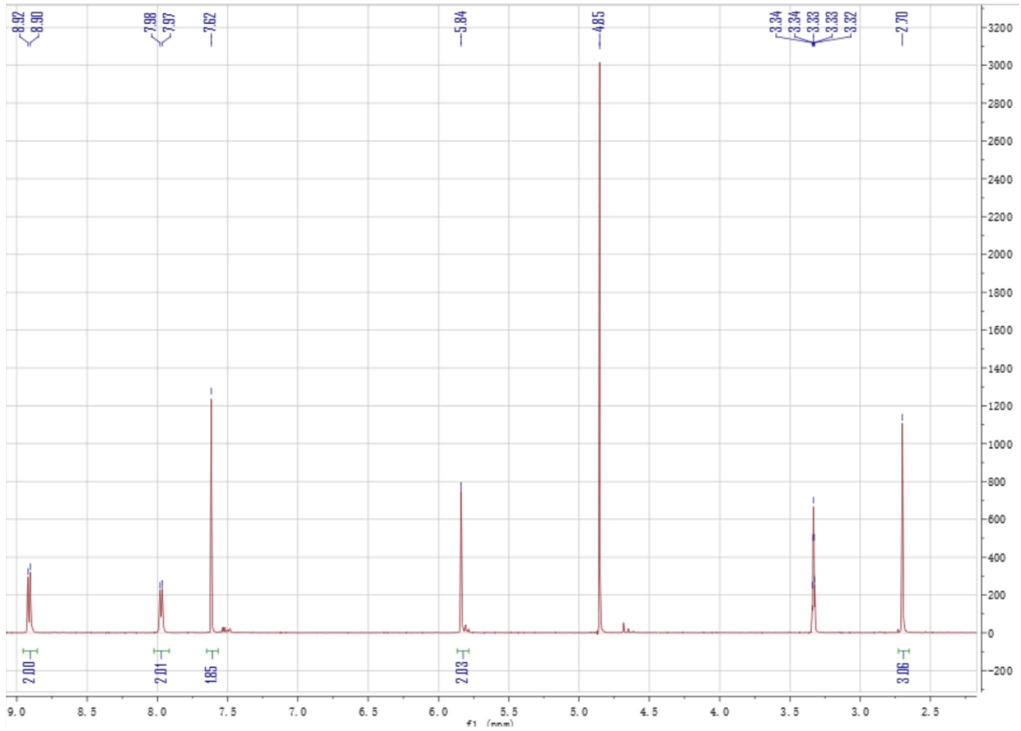

Figure S1 ^{1}H NMR of PY-BC in CD_3OD.

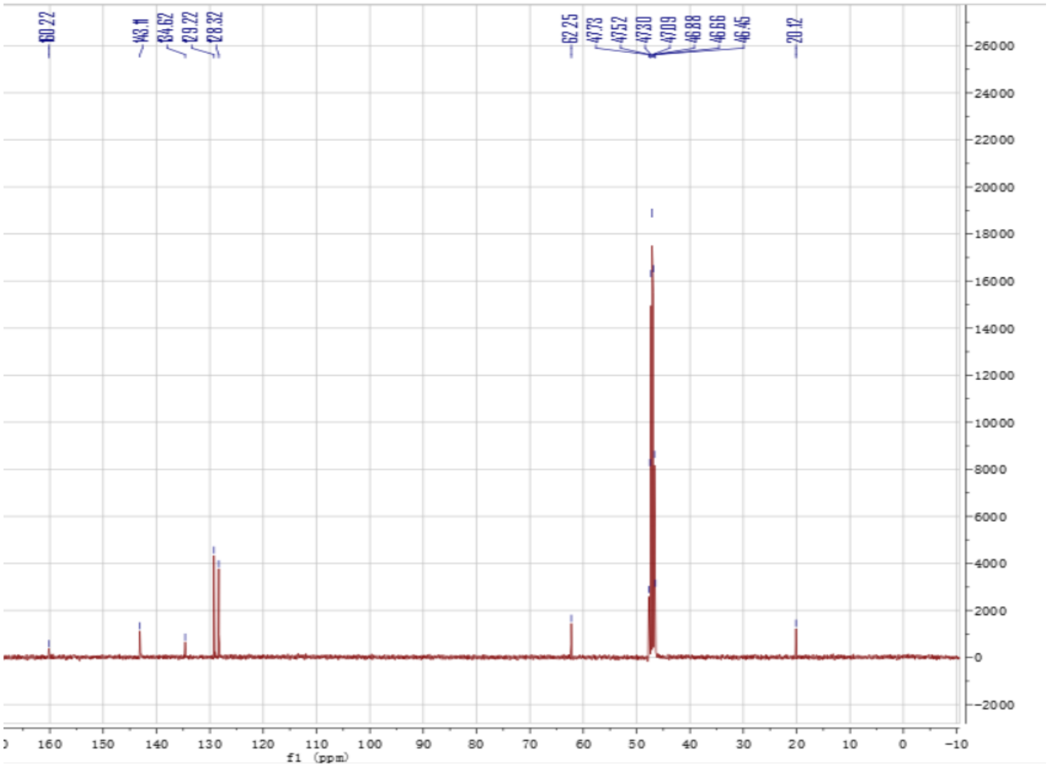

Figure S2 ^{13}C NMR of PY-BC in CD_3OD.

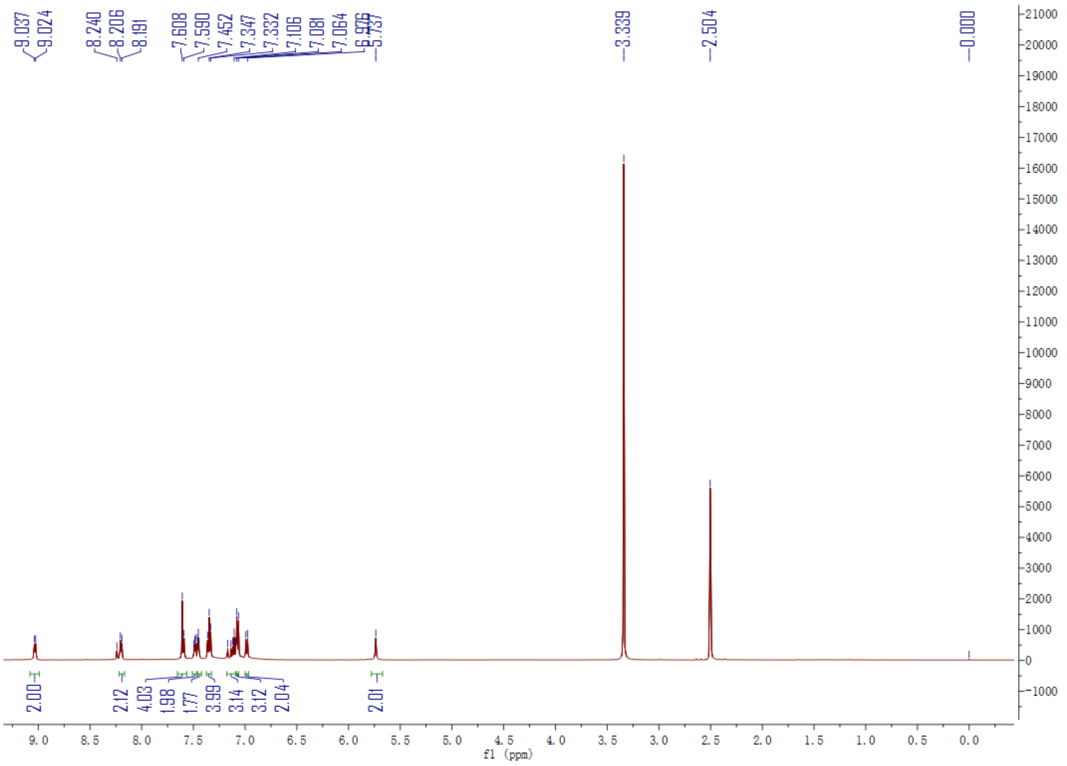

Figure S3 ^{1}H NMR of DTTPB in DMSO-d_6.

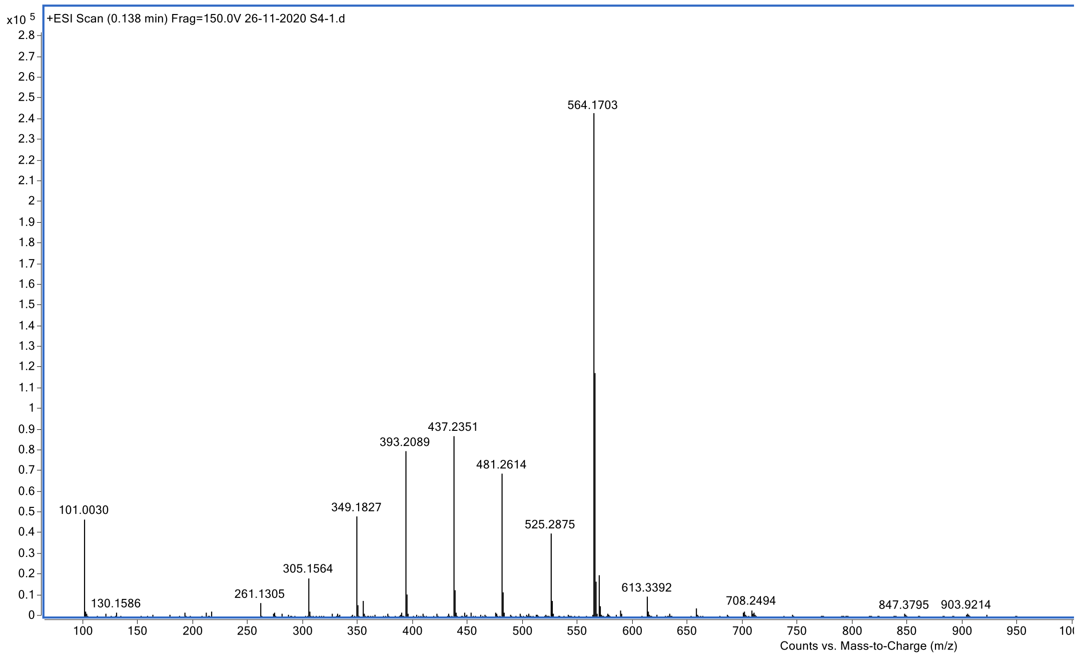

Figure S4 HRMS of DTTPB.

3. Supplementary Figures

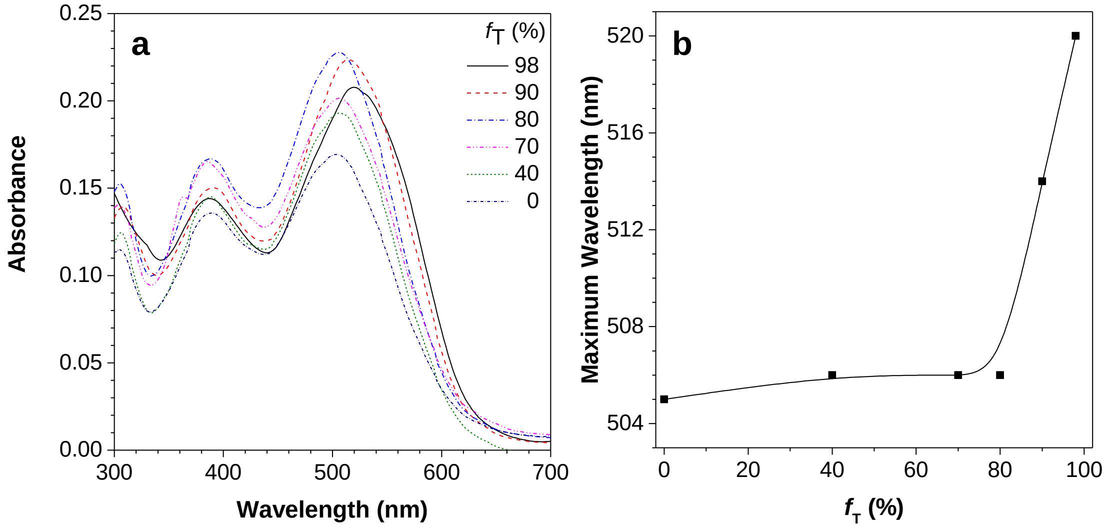

Figure S5. (a) Absorption spectra of DTTPB (2.5 μM) in the solvent mixtures of DMSO and toluene with different toluene fractions (f_T). (b) Maximum absorption wavelength changes in the solvent mixtures of DMSO and toluene with f_T.

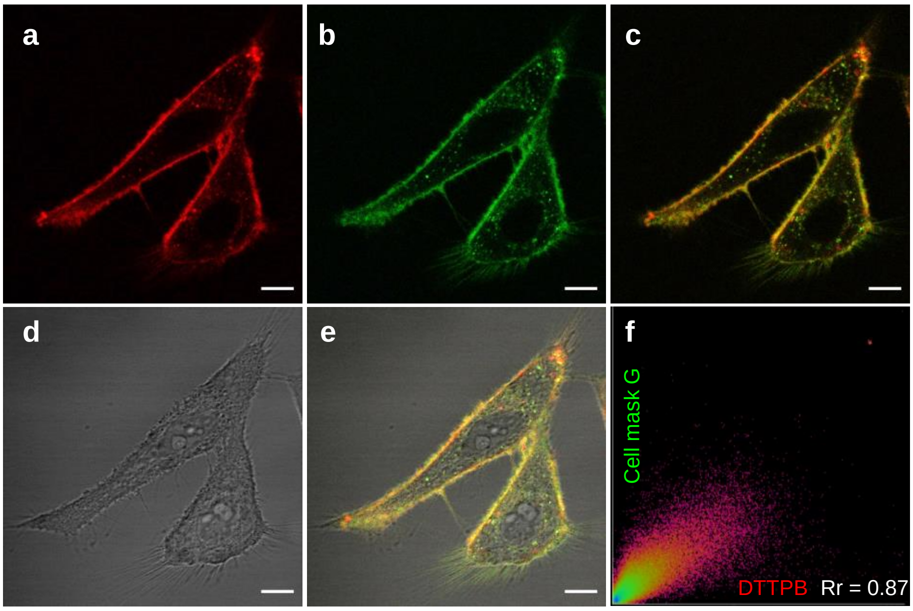

Figure S6. Plasma membrane co-stained with DTTPB and CellMask™ Green in HeLa cells. The plasma membranes of HeLa cells were stained with (a) DTTPB (red), (b) CellMask™ Green (green), (c) overlaid image of (a) and (b), (d) bright-field and (e) overlaid image of all four channels. Scale bar: 10 μm. (f) The scatter plot of the two channels with a Pearson correlation coefficient of 0.87.

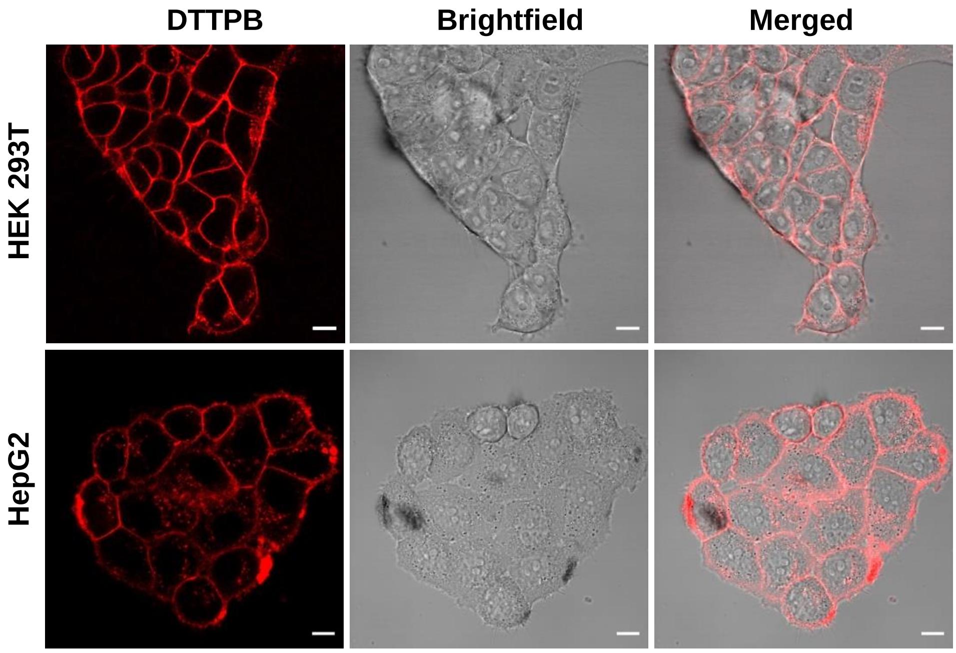

Figure S7. Images of plasma membrane in HEK293T and HepG2 cells stained with DTTPB (5 μM). Scale bar: 10 μm.

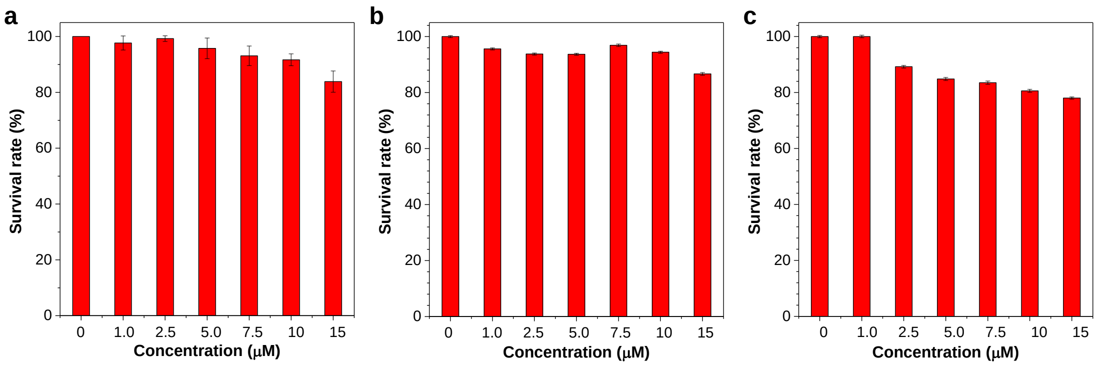

Figure S8. Cytotoxicity evaluated by CCK-8 assay in (a) NIH 3T3, (b) BHK-21 and (c) MRC-5 cells after incubation with different concentrations of DTTPB (0, 1, 2.5, 5, 7.5, 10 μM) for 24 h.

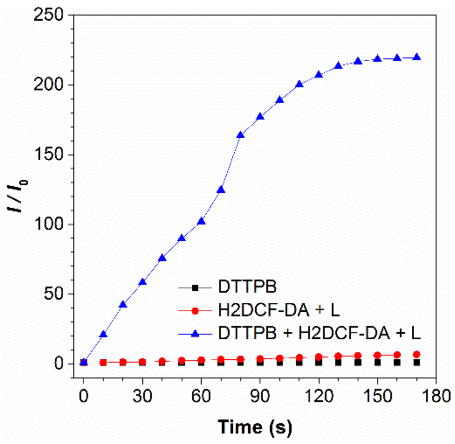

Figure S9. ROS sensitizing study of DTTPB upon white light irradiation. Relative changes in fluorescence intensity (I/I_0) at 534 nm of DTTPB (2 μM), H2DCF-DA (10 μM), mixtures of H2DCF-DA and DTTPB in PBS with white light irradiation for different times.

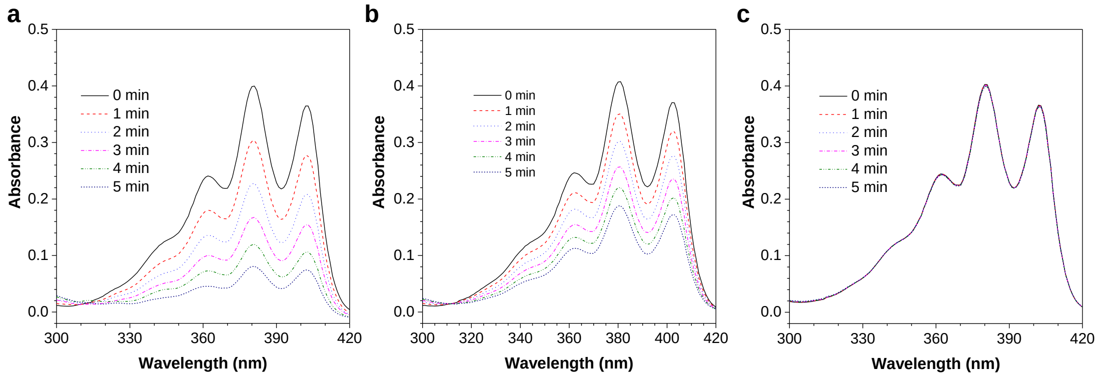

Figure S10. $^1O_2$ generation efficiency of DTTPB (2 μM) upon white-light irradiation. UV/Vis spectra of ABDA (50 mM) in the presence of (a) DTTPB, (b) RB and (c) blank control under white-light irradiation.

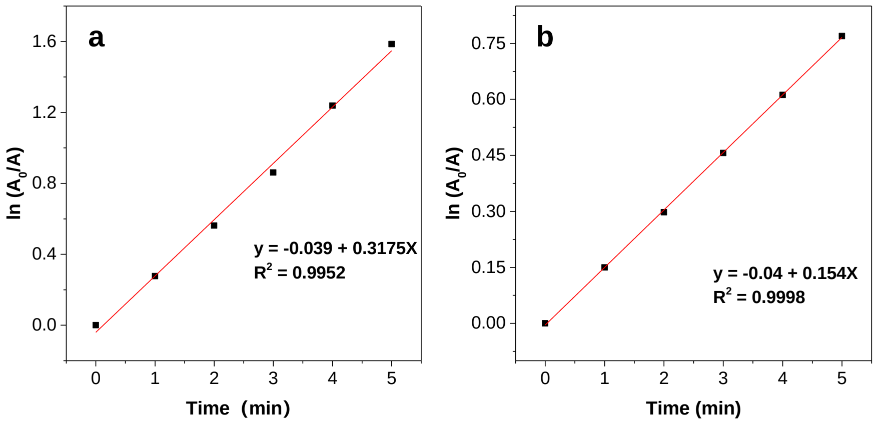

Figure S11. Decomposition rates of ABDA in the absence or presence of (a) DTTPB, and (b) RB under white-light irradiation, where A_0 and A are the initial and final absorbance of ABDA at 378 nm, respectively.

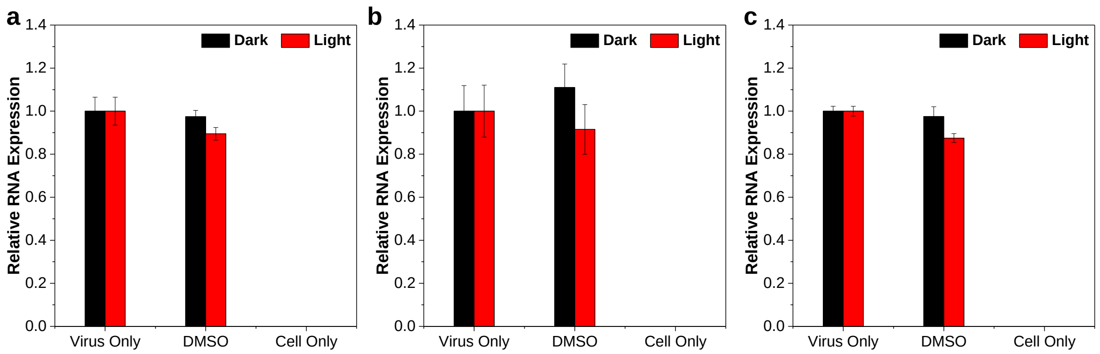

Figure S12. qPCR control experiments for the replication of the virus, (a) FMDV, (b) HCoV-OC43 and (c) HCoV-229E. Light: 9 mW/cm^2, white-light irradiation for 20 min, DMSO: 0.2% of culture medium (v/v). Data were expressed as mean ± SE, number of duplicates: 3.

Table S1. The qPCR primers used for detecting the RNA of FMDV, HCoV-OC43 and HCoV-229E.

qPCR primers	Fp（5'to 3'）	Rp（5'to 3'）
**FMDV(3D)**	GAACACATTCTTTACACCAGGAT	CATATCTTTGCCAATCAACATCAG
**HCoV-OC43**	AGCGTGGTTTTCTTGACAGG	TCTCAACAATGCGGTGTCCA
**HCoV-229E**	GGCAAACGGGTGGATTTGTC	CGCCTAACACCGTAACCTGT
**GAPDH**	CCACTCCTCCACCTTTGAC	ACCCTGTTGCTGTAGCCA

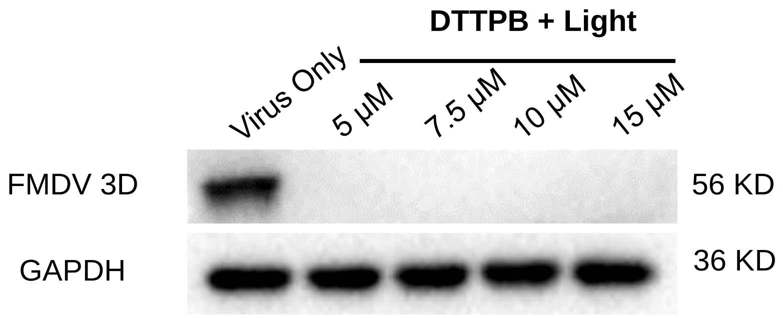

Figure S13. Western blot study of BHK-21 with FMDV infection after treated with different concentrations of DTTPB (5.0, 7.5, 10 and 15 μM) for 10 min and then irradiation with 9 mW/cm^2 white light for 20 min.

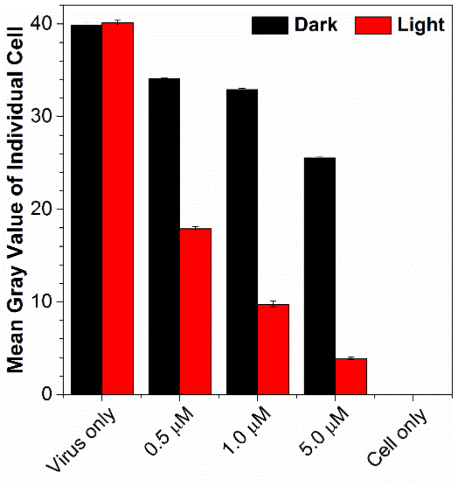

Figure S14. Mean of grey values in immunofluorescence images of MRC-5 cells after infection with DTTPB-treated with HCoV-OC43. The HCoV-OC43 were pretreated with different concentrations of DTTPB for 10 min and then irradiated with white light for 20 min (9 mW/cm^2). Data were expressed as mean ± SE, number of duplicates: 3.

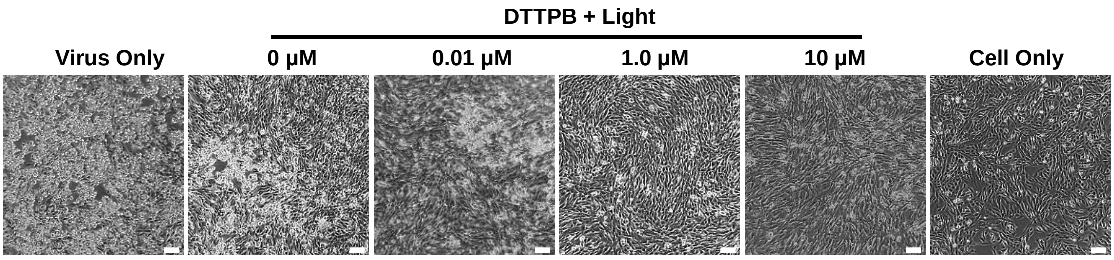

Figure S15. Morphology study of BHK-21 cells infected with DTTPB-treated-FMDV. Prior to infection of BHK-21 cells, FMDV were incubated with different concentrations of DTTPB for 10 min and then irradiated with white light for 20 min (9 mW/cm^2). The morphological changes were observed with microscope after 24 h

incubation of BHK-21 cells with FMDV. Scale bar: 50 μm.

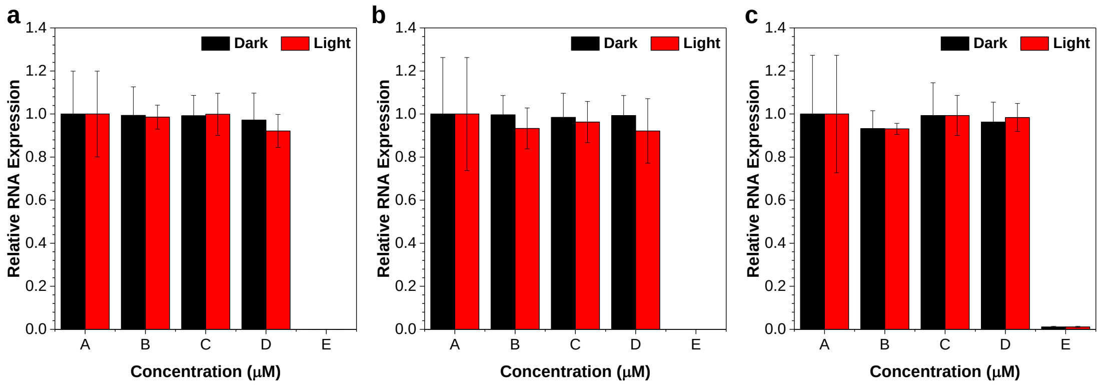

Figure S16. qPCR control experiments for the replication of (a) FMDV, (b) HCoV-OC43 and (c) HCoV-229E. Viruses were irradiated with 9 mW/cm^2 of white light for 20 min. DMSO volume fractions in the experiments: 0.2%. Data were expressed as mean ± SE, number of duplicates: 3. A: virus only, B: filter paper, C: filter paper + ethanol (air-drying), D: filter paper + ethanol + DMSO (air-drying), E: Cell only.
